# Patterning Biological Gels for 3D Cell Culture inside Microfluidic Devices by Local Surface Modification through Laminar Flow Patterning

**DOI:** 10.3390/mi11121112

**Published:** 2020-12-16

**Authors:** Joshua Loessberg-Zahl, Jelle Beumer, Albert van den Berg, Jan C. T. Eijkel, Andries D. van der Meer

**Affiliations:** 1 BIOS/Lab on a Chip, University of Twente, 7500-AE Enschede, The Netherlands; jellebeumer@gmail.com (J.B.); a.vandenberg@utwente.nl (A.v.d.B.); j.c.t.eijkel@utwente.nl (J.C.T.E.); 2 Applied Stem Cell Technologies, University of Twente, 7500-AE Enschede, The Netherlands; andries.vandermeer@utwente.nl

**Keywords:** 3D cell culture, microfluidics, hydrogel, cell migration, surface patterning, fabrication

## Abstract

Microfluidic devices are used extensively in the development of new in vitro cell culture models like organs-on-chips. A typical feature of such devices is the patterning of biological hydrogels to offer cultured cells and tissues a controlled three-dimensional microenvironment. A key challenge of hydrogel patterning is ensuring geometrical confinement of the gel, which is generally solved by inclusion of micropillars or phaseguides in the channels. Both of these methods often require costly cleanroom fabrication, which needs to be repeated even when only small changes need be made to the gel geometry, and inadvertently expose cultured cells to non-physiological and mechanically stiff structures. Here, we present a technique for facile patterning of hydrogel geometries in microfluidic chips, but without the need for any confining geometry built into the channel. Core to the technique is the use of laminar flow patterning to create a hydrophilic path through an otherwise hydrophobic microfluidic channel. When a liquid hydrogel is injected into the hydrophilic region, it is confined to this path by the surrounding hydrophobic regions. The various surface patterns that are enabled by laminar flow patterning can thereby be rendered into three-dimensional hydrogel structures. We demonstrate that the technique can be used in many different channel geometries while still giving the user control of key geometric parameters of the final hydrogel. Moreover, we show that human umbilical vein endothelial cells can be cultured for multiple days inside the devices with the patterned hydrogels and that they can be stimulated to migrate into the gel under the influence of trans-gel flows. Finally, we demonstrate that the patterned gels can withstand trans-gel flow velocities in excess of physiological interstitial flow velocities without rupturing or detaching. This novel hydrogel-patterning technique addresses fundamental challenges of existing methods for hydrogel patterning inside microfluidic chips, and can therefore be applied to improve design time and the physiological realism of microfluidic cell culture assays and organs-on-chips.

## 1. Introduction

**Figure 1:**
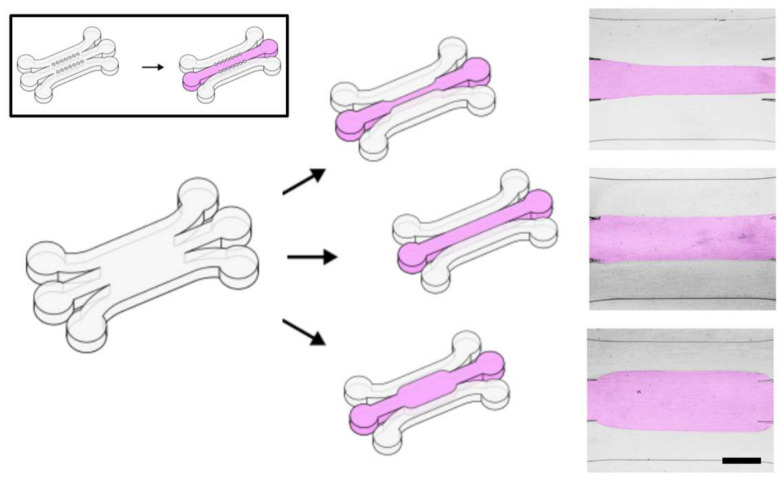
Local control of hydrophilicity allows patterning of biological hydrogels inside microfluidic devices without any geometrical constraints. Left: Schematic of 3-inlet, 3-outlet microfluidic devices. Empty channels are shown in white and gel-filled channels are shown in pink. Our devices have no confining geometry and therefore many different gel geometries are achievable in a single device geometry. Inset shows typical device found in the literature where geometry of the gel-filled region is fully determined by the device geometry, in this case with rows of micropillars. Right shows examples of 3 gels with geometry produced by our technique. Collagen gel is artificially colored pink for contrast. Scale bar is 0.5 mm.

Microfluidic devices are increasingly used to set up in vitro cell culture models of human tissues, either by parallelized culturing of cells in microfluidic microwells [1], or by culturing multiple cell types in a physiological microenvironment as in organs-on-chips [2,3]. In such devices, partially supported hydrogels are commonly used to offer cells a controlled three-dimensional (3D) culture environment [4,5,6]. The soft and fragile biological hydrogels are often supported by the relatively rigid walls of some microfluidic enclosure on one or more sides, leaving the other sides accessible for cell seeding and nutrient delivery. One of the most common designs is a gel-filled region bordered on two sides by fluidic access channels (Figure 1) [6,7,8,9,10,11,12,13,14]. This geometry in particular has found a wide range of applications as it allows both perfusion of the gel [7,8,9] and chemical gradient generation [10,11,12,13,14]. 

The geometry of the gel-filled region is key in determining the culture conditions therein. When specific chemical gradients are required, the width of the gel directly determines the steepness of the gradient. Biological hydrogels can also permit small fluid flows at low pressures due to their high hydraulic permeabilities (~5 × 10^−10^ cm^2^) relative to other hydrogels [15,16]. Similar to the case of chemical gradients, the width of the gel-filled region often determines its flow resistance and thus the shear experienced by cells in the gel for a given applied trans-gel pressure. Consequently, design flexibility in the gel geometry and particularly its width is highly desirable for precise definition of the cell culture conditions therein. 

Current microfluidic devices with side-by-side sandwiched hydrogels do not allow the geometry of the gel to be changed, because the geometry of the gel-filled region is completely defined by the geometry of the surrounding device. Most commonly, gel confinement is achieved via an array of pillars [12,13], or sometimes via phase guides [14,17]. In both cases, even small changes to the gel geometry require revision of these confining features. This takes extra time in the best case, but as devices are often molded from cleanroom-processed wafers, redesign can often be quite expensive, requiring the procurement of new lithography masks and extra cleanroom time.

Any geometric features used to confine the gel can also influence cells cultured on the gels in undesirable ways. Cell types cultured in monolayers on the gel-media interface are often known to have strong interactions with stiff and rough surfaces [18,19]. Proximity to protruding features on the surface of the microfluidic device used to keep the gel in place, particularly pillars, can therefore affect cell phenotype. When pillars are used to confine the gel, cells often have trouble bridging the gap between pillars and gel, and instead creep along the boundary between the two. This can make the monolayer unduly rough, and in the worst case, leave it leaky with large intrusions of the monolayer into the gel [11,12,13]. Integrity of the monolayer is particularly important, as the cell monolayer is often being studied directly or is included to ensure that physiological levels of nutrients or growth factors reach cells in the bulk of the gel [4,5,6].

The technique we present here allows the creation of microfluidic devices with a gel-filled region between two fluidic access channels, without the need for any confining geometry. This is achieved via local surface modification via laminar flow patterning. We show that the technique allows the easy adjustment of the width and shape of the gel-filled region and we demonstrate successful culture of endothelial monolayers on the gels. Our technique allows direct interaction between cells and hydrogels without obtruding structures in the region of interest, and will thereby aid in the engineering of organs-on-chips with more physiologically relevant microenvironments.

## 2. Results and Discussion

### 2.1. Laminar Flow Patterning of Surface Hydrophilicity

Our technique for patterning biological hydrogels uses a standard 3-inlet, 3-outlet microfluidic device, as with related techniques, but without the need for guiding micropillar or phase guide geometries (Figure 1). We achieve patterning of the hydrogel by specifically creating a hydrophilic path through an otherwise hydrophobic device [20,21,22]. When a hydrogel of choice is injected into the device, it stays confined to this hydrophilic path while it cures.

Before performing the patterning technique, a pre-treatment is applied to all surfaces in the device to render them hydrophobic and reactive to proteins. Both hydrophobicity and strong protein adhesion were achieved by sequential pre-treatments with (3-Aminopropyl) triethoxysilane (APTES) and glutaraldehyde (GA). GA will bind any protein with a free primary amine. In this study, we were working with PDMS (Polydimethylsiloxane) devices, but the same techniques should work for glass devices [23] and alternative chemistries do exist for plastics [24,25]. Specifics of our pre-treatment chemistry are discussed briefly at the end of this section.

**Figure 2:**
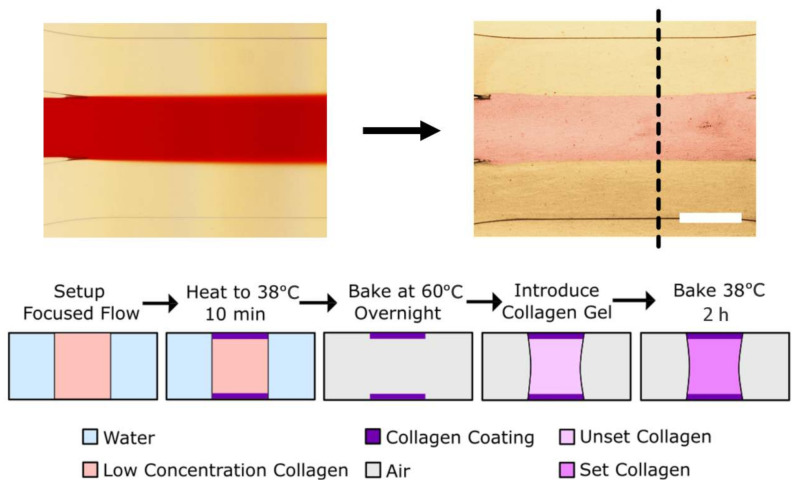
We show the process for selectively patterning a hydrophilic path in the middle of a channel via laminar flow patterning, and show that a cured hydrogel is easily confined to this pre-defined area. In the top left, a stream containing 10 µg/mL of collagen is confined to the center of the channel by its neighboring flows. The stream is artificially colored red for contrast. Subsequently, as shown on the top right, a high concentration of collagen (artificially colored pink for contrast) is confined to the patterned area and has gelled in place. Bottom shows the step-by-step procedure in schematic cross-section. Sections taken at dashed line in the top right image. Scale bar is 0.5 mm.

Following the pre-treatment, hydrophilic regions are patterned by introducing a low concentration solution of collagen I (10 µg/mL) into regions desired to be hydrophilic via laminar flow focusing (Figure 2) [26]. In the case shown in Figure 2 here, equal flows are driven through the three inlets on one side on the main channel by a syringe pump, and liquid is allowed to flow out the three outlets on the other side. The middle stream, dyed red with food coloring for easy visualization in Figure 2, normally contains the coating solution. The collagen covalently binds to the surface and, even after thorough drying and rinsing, renders the surface hydrophilic everywhere where it had contact. Flow was maintained for 10 min, before air-drying the channel. While the surface patterns achieved here were created via laminar flow patterning, other common techniques for creating micro surface patterns [27,28,29] should also work and may be tried in future work.

After patterning, the top and bottom of what will become the gel filled region have been rendered hydrophilic while the rest of the device remains hydrophobic. Through the middle inlet, a highly concentrated solution of collagen I (4 mg/mL) is then introduced. While the uncured gel solution is free to easily wet the hydrophilic regions of the device, it pins on the edge between the hydrophilic and hydrophobic regions. After gel filling, the devices are incubated to cure the gel and the fluidic access channels are filled with cell media (Figure 2).

The pre-treatment chemistry used here achieves two goals. First, it ensures that regions of the device can be rendered hydrophilic or hydrophobic, and second, it ensures strong adhesion between the used hydrogel and the walls of the channel (Figure 2). Similar pre-treatments are commonly used in some protein-based sensor technologies; however, they have stringent requirements on the thickness of the adsorbed layer of (3-Aminopropyl)triethoxysilane (APTES) and GA, as it can affect the performance of the sensor [30]. As such, those experimenters are required to grow their layers slowly, often with cumbersome vapor deposition techniques. For our application, this limitation is absent, as all we require is that the surface is hydrophobic and has many sites capable of binding proteins. Our protocol thus differs from the literature protocols. Specifically, all surface treatments in our protocol are done in aqueous phase for ease of use and at relatively high concentrations, reducing the reaction time from hours to minutes.

It is worth noting that the presented technique should be able to confine any liquid with a similar hydrophilicity to water, the only major constraint being that gels must be able to be injected as a liquid and then cured in chip. The collagen gel we use has a slightly higher than typical concentration, so to demonstrate that the technique works with less viscous liquids as well, we tested the technique with water and found the water similarly confined to the patterned region. (Shown in Figure S1).

### 2.2. Gel Geometry Control by Flow and Device Geometry

**Figure 3:**
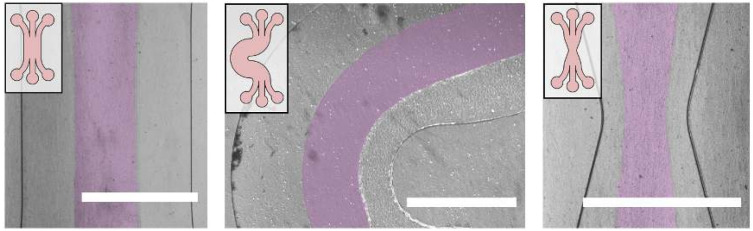
Gel geometries are determined by a combination of device geometry and flow parameters. In all micrographs, the gel-filled region has been artificially colored pink for clarity. Patterning in different devices yield different gel geometries that follow the contours of the channels. Insets show device designs and the full panels show the resulting gel geometries. Scale bars are 1 mm.

We patterned gels in microfluidic devices with three different types of geometries to show the ability of our technique to create both typical gel geometries and new, potentially useful gel geometries (Figure 3). In the simplest case (Figure 3, left) we show that our technique can realize the typical sandwiched gel design often used for generating simple gradients [10,11,12,13,14]. In Figure 3, middle, we show our ability to fabricate long, highly curved structures. The meandering channel shown in Figure 3 is 2 cm in length and the patterned gel maintains an effective barrier between the two fluidic access channels for the entire length of the device. Finally, with the tapered “hourglass” gel pattern shown in Figure 3, right, we show that our technique can also capture geometries with varying gel widths, which are typically used to generate many different gradients in the same device [31].

The smooth interfaces shown here are difficult to achieve in the commonly used pillared devices, where the gel-media interface arcs from pillar to pillar [12,13]. However, there are still some design constraints. In particular, the gel can only follow paths patternable via laminar flow patterning. Drawbacks of laminar flow patterning, like the propensity of flow to cut tight corners can cause some distortion of the patterned geometry (Figure 3, middle) and must therefore be accounted for. 

Gel geometries could also be varied by flow rate control. To control the width of the final gel, the flow rate of the central stream was varied during patterning while the flow rate of the side streams was left the same. This is shown in Figure 1, right. In these devices, the fraction of the total channel width patterned can be estimated simply as the ratio of the patterning stream’s flowrate to the total flowrate [32]. The gel widths shown in Figure 1 were generated with flow rate ratios of, from top to bottom, 1:0.5:1, 1:1:1, and 1:2:1. The minimum width of gel that we could reliably generate was 200 μm. It is worth noting that, due to the slow diffusivity of collagen and our ability to set a relatively fast flowrate, we do not see any broadening of the profile along the channel length as may occur due to diffusion of the patterning solution.

As the width of the patterned area is decreased, it becomes increasingly difficult to fill the chip by pipetting. Small changes in the volume from sticking and slipping of the pipette plunger cause the filling front to leap quickly forward in the device. It is during these forward leaps that the injected gel tends to burst out of the patterned area. Increasing care was required for narrower and narrower patterns, so we therefore postulate that filling the device with a steadier flow (i.e., flow generated by a tightly regulated pressure) during injection could allow for a smaller final width. 

Along similar lines, the strength of the capillary barrier should decrease with increased height of the device. Working with smaller channel heights should result in a higher Laplace pressure and thus stronger capillary barrier and could help realize narrower gel geometry. 

### 2.3. Cell Culture on Patterned Gels

**Figure 4:**
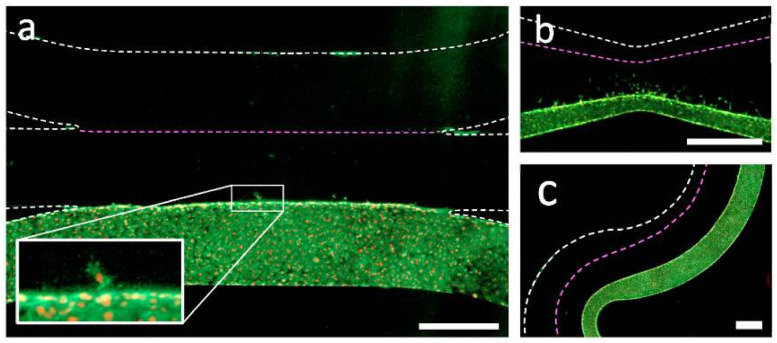
Human endothelial cells are confined by the patterned gel geometries and migrate into the gels. (**a**) Simple 3-channel geometry with patterned hydrogel in the center after 5 days of culturing. Endothelial cells show normal F-actin (green) and nuclear (red) staining. Few cells have spontaneously migrated into the gel (inset). (**b**) Cells grown in a device with an hourglass geometry. (**c**) Cells grown in a meandering channel. The boundary between the gel and the non-cultured fluidic access channel is indicated by the dotted pink lines, and the dotted white lines indicate the walls of the microfluidic channel. Scale bars are 500 microns.

To highlight the biocompatibility of the devices manufactured with the technique, we cultured human umbilical vein endothelial cells (HUVEC) on our gels. After 5 days of culturing, the HUVEC exhibit their characteristic cobble-stone morphology and remain attached to the substrate in all cases (Figure 4 and Figure S2). The adhesion of cells to the gel-media interface was confirmed by confocal imaging (Figure S3). The patterned geometries also remained unchanged by the cultured cells for the 5 days over which we observed the devices, indicating that the gel has remained strongly bound to the walls. Finally, some HUVEC spontaneously migrated into the collagen gels, even in absence of a chemotactic gradient (Figure 4a,b). The observed variation in cell migration from device to device is thereby most likely due to differences in seeding density.

### 2.4. Cell Migration through Patterned Gels

**Figure 5:**
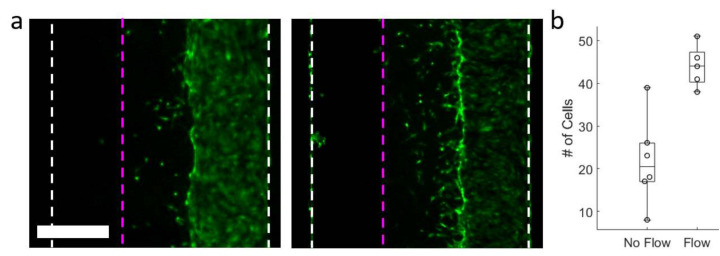
Endothelial cells migrate into patterned gels when trans-gel flow patterns are applied. (**a**) Green Fluorescent Protein (GFP)-expressing human umbilical vein endothelial cells (HUVEC) to small extent migrate into gels without any physical cues (**left**), but migrate significantly more when they are subjected to a trans-gel flow (**right**). The boundary between the gel and the non-cultured fluidic access channel is indicated by the dotted pink lines and the dotted white lines indicate the walls of the microfluidic channel. Scale bar is 0.5 mm. (**b**) Quantification of migration data shows a significant difference between conditions of no trans-gel flow and with trans-gel flow (*p* < 0.05). All data points are shown while boxes and whiskers denote data quartiles.

We also ran model experiments to measure cell migration in response to interstitial flow through the patterned gels as both a stress test of the stability of the patterned gels and a further proof of biocompatibility. HUVEC were used in our proof of concept as they are known to migrate in the upstream direction when interstitial flow is applied [7,8,9]. We cultured Green Fluorescent Protein (GFP) expressing HUVEC in one fluidic access channel and applied a hydrostatic pressure (~1.5 cm water) to the cell-free channel to induce trans-gel flow one day after seeding. While the flows we applied were several times higher than interstitial flows expected under normal physiological conditions (50 µm/s in our case), an effect on the cell migration was still apparent (Figure 5a). We counted the number of cells that had migrated from the seeded channel into the gel region on day 4, and found that the devices in which flow had been applied showed significantly higher counts, with a *p* value of 0.002 (Figure 5b).

Importantly, the gels also showed no sign of collapse or detachment in spite of the fact that trans-gel flow was significantly in excess of the physiological range. The stress on the gel and therefore damage to the gel is expected to increase with extra flow [33]. The fact that no damage was seen at these high flow rates implies that experiments executed within the physiological range of flow rates will leave the gel similarly unscathed. 

## 3. Conclusions and Outlook

We have designed and tested a new technique for making defined gel structures for 3D cell culture in microfluidic devices. Our technique can reproduce the most commonly used gel structures, but differs from existing techniques in that it uses laminar flow patterning to define the gel geometry instead of confining structures like pillars or phase guides. This both allows rapid prototyping of the gel geometry and reduces contact between cells and the unnaturally stiff walls of the device.

We have shown that the technique is usable to achieve a variety of geometries, both common in the literature and novel. This includes devices with gels far longer and with greater curvature than what is commonly used in the literature, showing that our technique meets and exceeds existing geometric requirements. We demonstrate that the width of the gel is easily controlled by adjusting flow parameters during patterning. Once gels are patterned and cured, human cells can be grown in the devices and their migratory behavior can be studied. Finally, we show that devices can withstand trans-gel pressures far in excess of what is needed to reproduce physiological interstitial flow velocities. Taken together, the features provided by this technique should make it a powerful tool for fast implementation and iteration of 3D cell culture devices. 

## 4. Methods

### 4.1. PDMS Device Fabrication

To fabricate the PDMS devices, un-cured PDMS was cast on a silicon mold with photopatterned SU8 (MicroChem, Round Rock, TX, USA) microstructures. SU8 features were 100 micrometers high. Un-cured PDMS (Sylgard 184) was prepared at a 10:1 polymer to cross linker ratio. The devices were then heated overnight at 60 °C to cure the polymer before demolding. In parallel, microscope slides dipped in the same PDMS were prepared as a substrate for bonding. Coated slides were similarly cured overnight at 60 °C. After demolding, inlets were punched using a 1 mm Harris Uni-Core biopsy punch (Ted Pella Inc., Redding, CA, USA). Both the cast devices and PDMS coated slides were then exposed to oxygen plasma in a PDC-001 oxygen plasma cleaner (Harrick Plasma, Ithaca, NY, USA) to activate their surfaces. The cast polymer was then gently pressed onto the PDMS coated slides to form the completed PDMS devices.

### 4.2. Surface Treatment

To make the surface able to covalently bind proteins, sequential (3-Aminopropyl)triethoxysilane (APTES) and glutaraldehyde (GA) treatments were performed. This protocol was applied within 15 min after the plasma treatment described in the previous step. A 3% (*v*/*v*) solution of APTES (Sigma-Aldrich, St. Louis, MO, USA) in deionized water was first introduced into the devices and left to sit for 30 min. Filtered air was blown through the devices to dry them and the devices were flushed three times with deionized water. A 10% (*v*/*v*) solution of GA (Sigma-Aldrich) was then prepared in 1× phosphate buffered saline (PBS) (Sigma-Aldrich). This solution was similarly pipetted into the devices and allowed to sit for 30 min. Finally, the devices were again blown dry and rinsed three times with deionized water. Devices were then baked at 60 °C overnight to drive off any excess water or reactants.

### 4.3. Flow Patterning

To pattern the devices, a 10 μg/mL solution of collagen was first prepared. This was done by gently mixing rat tail collagen I (Corning, Corning, NY, USA) with cold 1× PBS. The solution was then loaded into a 1 mL syringe (Hamilton, Reno, NV, USA) and placed in a syringe pump (neMYSES) along with two deionized water containing 1 mL syringes. Tygon tubing (TYGON) was used to connect the syringes to the device. The middle inlet was connected to the collagen containing syringe while the two outside channels were connected to the deionized water containing syringes. Tygon tubing was also used to connect the outlets to a waste container. Tubing from the middle outlet was cut slightly shorter to ensure that the entire stream of coating solution flowed out this outlet. Finally, the pumps were used to drive flow through the devices with flow rates of 30 μm per minute each. The flow rates of deionized water streams were left constant while the flow rate of the collagen containing stream was adjusted to change the width of the patterned region depending on the desired gel region width. Flow was maintained for 10 min for complete patterning. Devices were then blown dry using filtered compressed air and baked overnight at 60 °C to dry completely.

### 4.4. Cell Culture

In general, HUVECs from Lonza (Catalog#: C2519A) were used. In the specific case of the interstitial flow experiments, GFP expressing HUVECs purchased form Cellworks (product code: ZHC-2402) were used for live imaging of the growing cultures. In all cases, cells were thawed at passage 5, and subcultured once in a coated t75 flask (CELLCOAT T75 flask, Greiner, Alphen aan den Rijn, the Netherlands) in endothelial growth medium (EGM; Sigma-Aldrich). Before chip seeding, HUVECs were trypsinized when they reached 80% confluency and cells were diluted to the appropriate concentration.

### 4.5. Device Filling, Cell Seeding, and Culture

A 4 mg/mL solution of rat tail collagen I was prepared at neutral pH on ice. This solution was gently pipetted into the central inlet of the patterned microfluidic devices and cured for 2 h at 37 °C. To fill the fluidic access channels, devices were submerged in degassed 1x PBS overnight. The next day, the fluidic access channels are filled with cell media (EGM) and seeded with a cell suspension at a concentration of 0.5 million cells/mL. The devices were tilted at an angle of 45° to allow the cells to settle directly onto the gel-media interface for 10 min after seeding. The cell suspension was then replaced with media to remove unattached cells. Subsequently, cell media was replaced every day during chip culture.

### 4.6. Cell Fixation, Staining, and Imaging

The cells were fixated with a 4% paraformaldehyde (Sigma Aldrich) solution then treated with a 0.3% Triton X-100 (Sigma Aldrich) solution in phosphate buffered saline to permeate them. The nuclei were then stained with NucBlue (ThermoFisher Scientific, Waltham, MA, USA) while F-actin filaments were stained with ActinGreen (ThermoFisher Scientific). In some figures, the blue channel has been shown as red to help more clearly visualize the cell nuclei. All fluorescence microscopy images of the cells were taken with an EVOS FL cell imaging system. Confocal microscopy was performed on a Nikon confocal A1 (Nikon Corporation, Tokyo, Japan).

### 4.7. Interstitial Flow Experiments

Devices were first seeded as described above. Flow through the gel was applied one day after seeding. In these experiments, the inlets were fitted with pipette tips as reservoirs. Reservoirs on the gel inlet and outlet were filled with PDMS to prevent outflow of media. Reservoirs connected to fluidic access channels were filled with cell media. Reservoirs connected to the cell containing fluidic access channel of the device were filled with less media than reservoirs on the other inlets leaving a height difference of ~1.5 cm. This height difference was refreshed every day when media was replaced. At day 4, the number of cells that had migrated into the gel were counted. This was done by first manually by determining the gel boundary, then identifying the number of individual cells that had migrated into the gel region of a 2 cm long section of channel. A student’s *t*-test was performed in Matlab (Mathworks, Natick, MA, USA) to test significance of the difference between devices with flow and devices with no flow, and a *p* of 0.002 was recovered.

## Supplementary Materials

The following are available online at https://www.mdpi.com/2072-666X/11/12/1112/s1, Figure S1: Patterned device filled with water instead of a collagen gel to demonstrate the ability to fill the devices with alternative liquids. Scalebar is 500 μm. Figure S2: Higher magnification image of HUVECSs in one of our devices showing the characteristic cobblestone pattern. Scalebar is 200 microns. The cells are stained as described in the Methods section. Figure S3: Confocal images of HUVECs cultured in our devices. In each image, the media channel is to the right and the gel is to the left. The left panel shows a cross section taken perpendicular to the gel-media interface and the right panel shows the same volume from another angle. The cells are stained as described in the methods section. It can be seen that the endothelial cells cover the gel-media interface and that some cells migrate into the gel. Scale bar is 50 μm.
