# Supplementary material for: Patterning Biological Gels for 3D Cell Culture inside Microfluidic Devices by Local Surface Modification through Laminar Flow Patterning
Source: Micromachines (Basel). 2020 Dec 16;11(12):1112. doi: 10.3390/mi11121112 (PMC7765499; doi:10.3390/mi11121112)
Supplement: Supplementary file 1 [file micromachines-11-01112-s001.pdf]

Supplementary Information

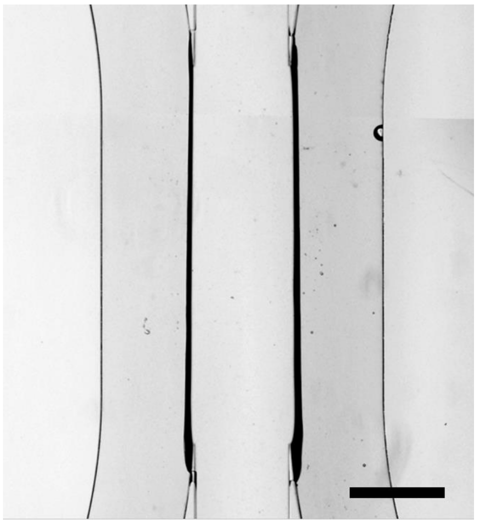

Figure S1. Patterned device filled with water instead of a collagen gel to demonstrate the ability to fill the devices with alternative liquids. Scalebar is 500 µm.

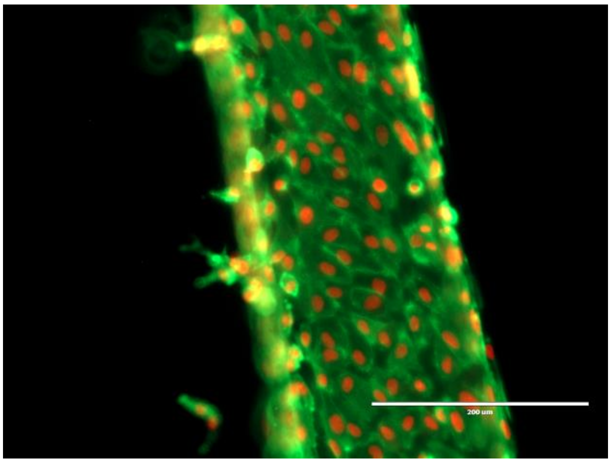

Figure S2. Higher magnification image of HUVECSs in one of our devices showing the characteristic cobblestone pattern. Scalebar is 200 µm. The cells are stained as described in the Methods section.

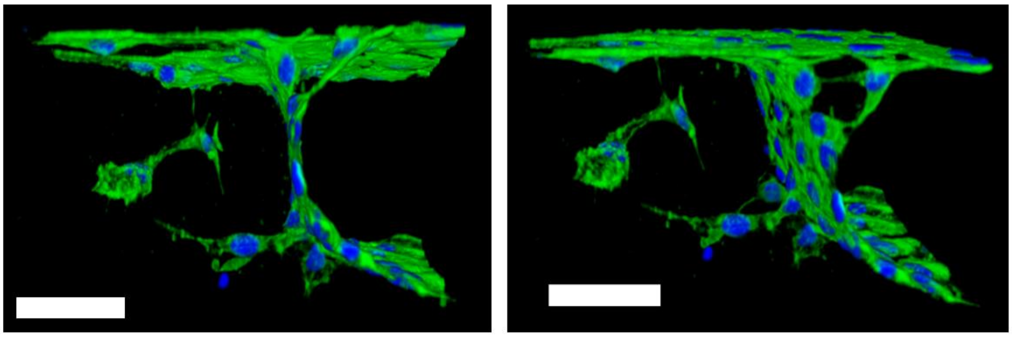

Figure S3. Confocal images of HUVECs cultured in our devices. In each image, the media channel is to the right and the gel is to the left. The left panel shows a cross section taken perpendicular to the gel-media interface and the right panel shows the same volume from another angle. The cells are stained as described in the methods section. It can be seen that the endothelial cells cover the gel-media interface and that some cells migrate into the gel. Scale bar is 50 µm.
